# Supplementary material for: The Discrepancy between Subjective and Objective Evaluations of Cognitive and Functional Ability among People with Schizophrenia: A Systematic Review
Source: Behav Sci (Basel). 2023 Dec 30;14(1):30. doi: 10.3390/bs14010030 (PMC10812940; doi:10.3390/bs14010030)

Table S1. Results of ROBUST Assessment.

Article	*Sampling Frame*	*Participant Recruitment*	*Exclusion Rate*	*Sample Size*	*Sample Characteristics*	*Measurement Validity*	*Setting*	*Data Management*	*Total*
Zanello & Huguelet (2001) [14]	1	0	0	0	1	0	1	0	3
Bowie et al. (2007) [26]	0	0	0	0	1	0	1	0	2
Medalia et al. (2008) [15]	1	0	1	0	1	0	1	0	4
Donohoe et al. (2009) [16]	1	0	0	0	1	0	1	0	3
Lecardeur et al. (2009) [17]	1	0	0	1	1	0	1	0	4
Johnson et al. (2011) [18]	1	0	0	1	1	0	1	0	4
Sabbag et al. (2011) [27]	1	0	0	1	1	0	1	0	4
Sabbag et al. (2012) [28]	1	0	1	1	0	0	1	1	5
Saperstein et al. (2012) [19]	1	0	1	0	1	1	1	0	5
Gould et al. (2013) [29]	1	0	0	1	1	0	1	0	4
Harvey et al. (2013) [30]	1	0	0	1	1	0	1	0	4
Sellwood et al. (2013) [20]	1	0	0	1	1	1	1	0	5
Durand et al. (2014) [38]	1	0	1	1	1	0	1	0	5
Chang et al. (2015) [21]	1	0	0	1	1	1	1	0	5
Gould et al. (2015) [2]	1	0	0	1	1	0	1	0	4
Prouteau et al. (2015) [22]	1	0	0	0	1	0	1	0	3

Harvey et al. (2017) [31]	1	0	0	1	1	0	1	1	5
Olsson et al. (2019) [32]	1	0	0	1	1	0	1	0	4
Harvey et al. (2019) [33]	1	0	0	1	1	0	1	0	4
Durand et al. (2021) [34]	1	0	0	1	1	1	1	0	5
Haugen et al. (2021) [24]	1	0	0	0	1	1	1	1	5
Rocca et al. (2021) [35]	1	0	1	1	1	0	1	0	5
Tercero et al. (2021) [23]	1	0	0	0	1	0	1	0	3
Jongs et al. (2022) [36]	1	0	1	0	1	0	1	0	4
Morgan et al. (2022) [25]	1	0	1	1	1	0	1	1	6
Okada (2022) [37]	1	0	0	1	0	0	1	0	3
Summary	96%	0%	27%	65%	92%	19%	100%	15%	4 (median)

1. Sampling frame: Is the sampling frame largely representative of the studied population?

   Yes (1) = they are identical, or the sampling frame is representative to a great extent

   No (0) = the sampling frame represents a much more specific group than the studied population or not reported

2. Participant recruitment: Were appropriate methods utilized for participant recruitment?

   Yes (1) = relatively low sampling bias (e.g., random selection or randomly addressing participants)

   No (0) = high potential for sampling bias (e.g., convenience sample) or not reported

3. Exclusion rate: Is the exclusion rate of participants acceptable?

   Yes (1) = below 20% of participants were excluded

   No (0) = more than 20% excluded or not reported

4. Sample size: Is the final sample size sufficient for the research design?

Yes (1) = equal to or above N = 100

No (0) = less than the chosen criterion or not reported

5. Sample characteristics: Are the basic demographic characteristics of the sample reported?

Yes (1) = both age and gender are reported

No (0) = one or both not reported

6. Measurement validity: Do the relevant measures have adequate reliability?

Yes (1) = all measures have acceptable reliability (e.g., $\alpha > 0.7$ for a scale with 7 or more items)

No (0) = reliability of one measure or more is less than the chosen criterion or not reported

7. Setting: Were the procedures included in the study conducted in a controlled setting?

Yes (1) = controlled environment (e.g., laboratory or university settings)

No (0) = uncontrolled environment (e.g., online) or not reported

8. Data management: Are data cleaning and management procedures reported and/or acceptable?

Yes (1) = Reporting of either missing data, outliers, or invalid responses

No (0) = all unreported or unusual procedures utilized

Figure S1. PRISMA Flow Chart.

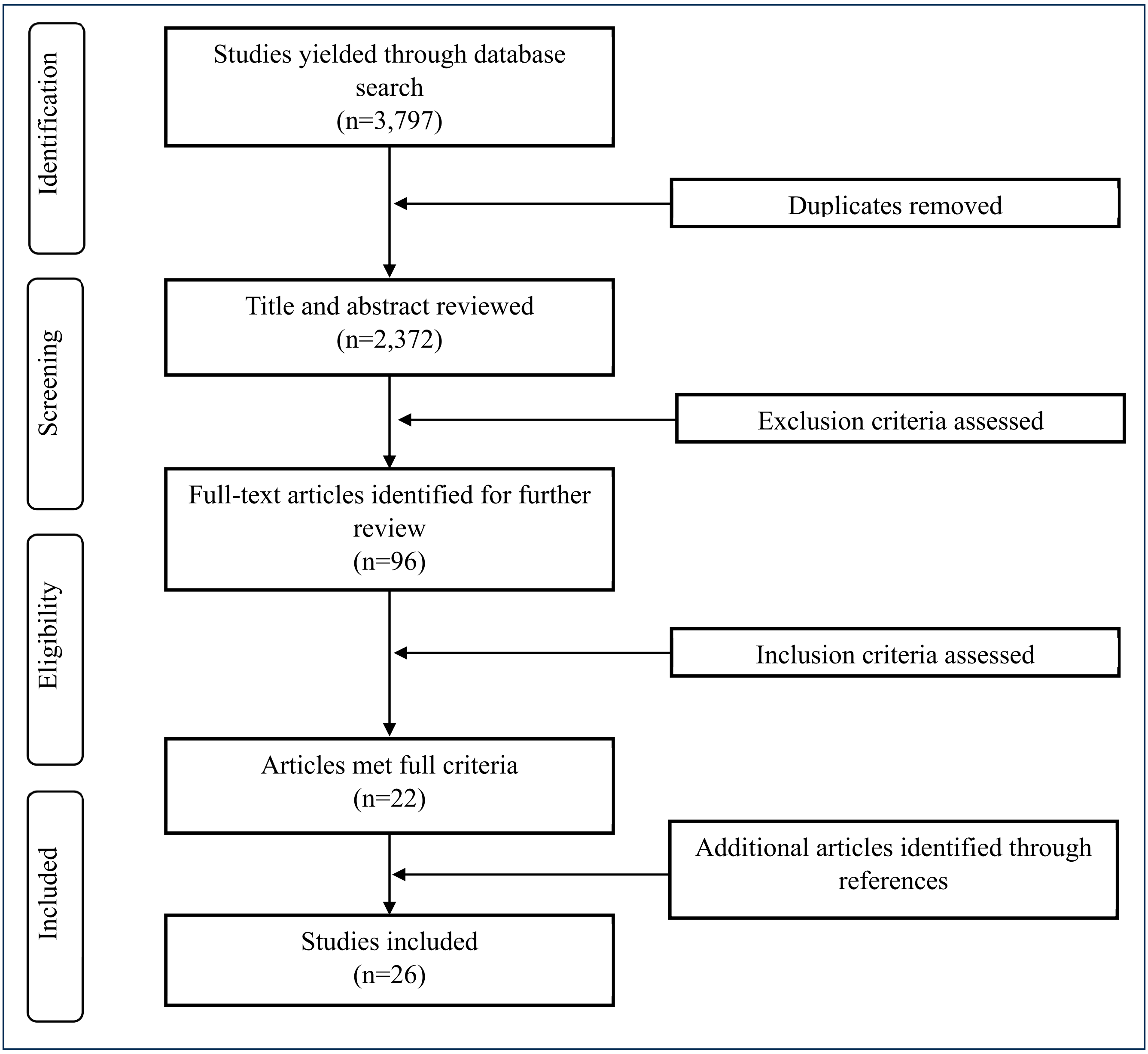

Supplement: Supplementary file 1 [file behavsci-14-00030-s001.zip › behavsci-2778554-supplementary.pdf]
